# Understanding Data-Driven Cyber-Physical-Social System (D-CPSS) Using a 7C Framework in Social Manufacturing Context

**DOI:** 10.3390/s20185319

**Published:** 2020-09-17

**Authors:** Dao Yin, Xinguo Ming, Xianyu Zhang

**Affiliations:** Institute of Industrial Engineering and Management, School of Mechanical Engineering, Shanghai Jiao Tong University, 800 Dongchuan Road, Minhang District, Shanghai 200240, China; yind_sjtu2013@sjtu.edu.cn (D.Y.); leitun@sjtu.edu.cn (X.Z.)

**Keywords:** data-driven, cyber-physical-social system, social manufacturing, cyber-physical system

## Abstract

The trend towards socialization, personalization and servitization in smart manufacturing has attracted the attention of researchers, practitioners and governments. Social manufacturing is a novel manufacturing paradigm responding to this trend. However, the current cyber–physical system (CPS) merges only cyber and physical space; social space is missing. A cyber–physical–social system (CPSS)-based smart manufacturing is in demand, which incorporates cyber space, physical space and social space. With the development of the Internet of Things and social networks, a large volume of data is generated. A data-driven view is necessary to link tri-space. However, there is a lack of systematical investigation on the integration of CPSS and the data-driven view in the context of social manufacturing. This article proposes a seven-layered framework for a data-driven CPSS (D-CPSS) along the data–information–knowledge–wisdom (DIKW) pyramid under a social manufacturing environment. The evolution, components, general model and framework of D-CPSS are illustrated. An illustrative example is provided to explain the proposed framework. Detailed discussion and future perspectives on implementation are also presented.

## 1. Introduction

In recent years, there is a trend of socialization, personalization and servitization in the manufacturing industry [1]. A new manufacturing paradigm, termed social manufacturing, has sprung up, which is concerned by collaborative, service-oriented, crowdsourcing and customer-centric industries [2]. Social issues in smart manufacturing have attracted the attention of researchers, practitioners and governments. In 2016, Japan launched the Society 5.0 initiative to solve social issues through a cyber–physical System (CPS) in the 5th Science and Technology Basic Plan [3].

CPS has gained intensive attention in recent years. The term CPS was firstly coined at American National Science Foundation (NSF) from an engineering perspective, in 2006, to describe the tight collaboration between the cyber world and physical world [4]. It demonstrates the integration between computation, communication and control. CPS was widely applied in the field of smart manufacturing, emergency response, air transportation, intelligent transportation, etc. [5]; however, most CPS applications failed to take the human factor into consideration as an internal element [6]. To this end, the cyber–physical–social system (CPSS) emerged [7], which was generally viewed as an extension of CPS, and seamlessly integrated cyber space, physical space and social space.

With the development of the Internet of Things and social networks, a large volume of data is generated from tri-space. How to handle heterogeneous data to support social manufacturing is a greatly challenging question. Existing studies on CPSS focused on either a generic model or specific application. Frameworks for D-CPSS were few, but did exist. Despite that [8] proposed a layered architecture for D-CPSS from a data centric perspective, the interactions among tri-space were not investigated. Social issues in the context of social manufacturing lacked exploration. Furthermore, a D-CPSS which integrates the interaction among tri-spaces for social manufacturing along the data–information–knowledge–wisdom (DIKW) pyramid has not yet been researched systematically. Lee and Bagheri [9] proposed a 5C (i.e. connection, conversion, cyber, cognition, and configuration) framework for CPS in the context of Industry 4.0, but social space was missing from this and it was not sufficient to support social manufacturing. Motivated by this, this paper aims to propose a 7C (i.e. connection, conversion, communication, computing, cognition, configuration, and collective intelligence) framework for better understanding D-CPSS under the social manufacturing environment.

The remainder of the paper is organized as follows: an overview of the literature on the evolution from CPS to CPSS, CPSS and social manufacturing are provided in Section 2. After presenting the change from 5C for CPS to 7C for CPSS and the description of D-CPSS, a 7C framework for D-CPSS in the social manufacturing context is proposed in Section 3. Section 4 provides an illustrative example to present the application of the proposed framework. Section 5 discusses the theoretical and practical implications, limitations and technical challenges of this paper. Section 6 concludes the article with proposed future research directions.

## 2. Related Work

This section reviews the related works on evolution from CPS to CPSS, CPSS and social manufacturing. A summary of the research gap is given.

### 2.1. From CPS to CPSS

**Figure 1:**
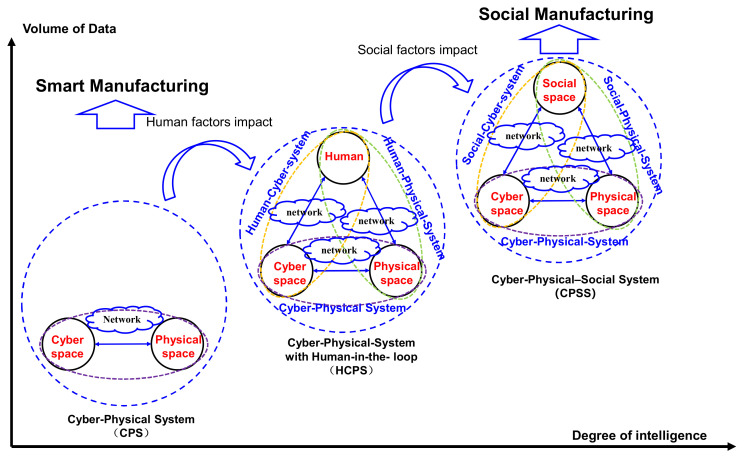
Evolution of the cyber–physical–social system (CPSS).

Figure 1 depicts the origin and development of CPSS. From the perspectives of intelligence and data volume, the evolution of CPSS can be divided into three stages, namely CPS, human–cyber–physical systems (HCPS) or human-in-the-loop cyber–physical systems (HiTLCPS), and CPSS.

On the first stage, CPS integrates computing, communication, and control by considering cyber space and physical space. CPS provides support for smart manufacturing. On the second stage, HCPS involves human factors. It contains three spaces, namely, human space, cyber space and physical space. The interactions among three spaces are supported by three systems, namely, the human–cyber system, CPS and human–physical system. On the third stage, based on HCPS, CPSS goes one step further by taking social factors into account. CPSS-based manufacturing aims to support social manufacturing. In these three stages, different spaces are connected via a network. From an evolution perspective, the detailed evolution path is introduced below.

(1) From CPS to HCPS.

The role of humans has changed from passive information receivers to information or knowledge generators [10]. Human tended to interact with enterprise throughout the product lifecycle. Previously, when interacting with CPS, humans played the role of passive recipients or passive consumers. To support social manufacturing, there was a need to involve human factors in the CPS architecture. Therefore, CPS evolved to HCPS. Zhou and Li [11] introduced the human–cyber–physical system (HCPS). Nunes and Zhang [6] conducted a survey on human-in-the-loop cyber–physical systems (HiTLCPS). Taking human interaction into consideration, [11] introduced HCPS and new-generation intelligent manufacturing, which was the integration of advanced manufacturing technology, new-generation artificial intelligence (AI) and social–technical systems.

(2) From HCPS to CPSS.

In previous literature, humans were limited to being individual actors, such as an operator in a manufacturing cell [12]. The power of the crowd was emphasized by researchers, including such topics as crowdsourcing, collaboration and collective intelligence. Social factors played more and more important roles in product development, manufacturing and services. CPSS-based social manufacturing was in demand, which extended human factors to social factors; HCPS moved to CPSS.

### 2.2. CPSS

Wang [13] first proposed the concept of CPSS and classified it into physical space, cyber space, the physical world, the mental world and the artificial world. Some researchers believed that CPSS was developed on the basis of CPS and the cyber–social system (CSS) [14,15,16]. Considering CPSS, researchers proposed different concepts, such as the cyber–physical–social system (CPSS), the social–cyber–physical system (SCPS), and the human–cyber–physical system (HCPS), as shown in Table 1. CPSS was typically used in energy, power grid, smart home, emergency management, smart vehicles, intelligent transportation system, intelligent manufacturing, and smart city, etc. [7].

Numerous scholars have investigated CPSS, which is commonly viewed as a system of systems integrating cyber, physical and social systems. For example, Bereket Abera and Yannick et al [4] reviewed the existing works on personalization in CPSS and defined CPSS as a system being composed of CPS, social systems, virtual space and physical space. Zeng and Yang [17] defined CPSS as the integration of CPS and the cyber–social system (CSS). Amit and Pramod [19] proposed physical–cyber–social (PCS) computing.

The existing literature on CPSS is mainly presented from the perspective of definition and concept, design, architecture and applications, as shown in Table 2.

Ning and Liu [18] proposed a novel concept—Cybermatics—and put forward a cyber–physical–social–thinking (CPST) hyperspace architecture integrating cyber services, physical objects, social people and human thinking. Marta and Angelink [20] employed a systematic mapping study to review the existing research of CPSS, including the definition, main characteristics and processes, architectural approaches to designing and describing CPSS, current research focuses and typical application scenarios. Yao and Lin [21] discussed the development of manufacturing paradigms and proposed socio–cyber–physical system (SCPS)-based manufacturing from the perspective of organizational semiotics. Wisdom manufacturing and social manufacturing were illustrated as two modes for SCPS-based manufacturing. CPS-based manufacturing was viewed as smart manufacturing, while SCPS-based manufacturing was viewed as wisdom manufacturing in some research [14].

As for the design of CPSS, Zeng and Yang [17] conducted a survey of system-level design methodologies and introduced the latest research advancement on design methodology for CPSS. Zeng and Yang [24] proposed a Petri net-based extended model for CPSS to meet the needs of the social scenario of multiple users. Jiang and Ding [1] proposed a CPSS framework based on CPS by adding the social aspect for a socialized production network (SPN). Yao and Zhou [14] presented a holistic survey of CPS, proposed the architecture of CPS-based smart manufacturing extended CPS-based manufacturing to social–CPS (SCPS)-based manufacturing, which was called wisdom manufacturing. However, these works ignored the important role of data flow throughout the cyber space, physical space, and social space.

As for the data-driven approaches and application in CPSS, Bin and Zhiwen [8] proposed a four-layered framework for data-centric cyber physical social systems (D-CPSS). De and Zhou [27] introduced the background and development of cyber–physical–social systems, including mechatronic systems, embedded systems, cyber–physical systems, cyber physical systems with human-in-the-loop, the Internet of Things and cyber–physical–social systems. A conceptual framework of CPSS was proposed from a data-centric perspective. The work [22,23] proposed a three-layer CPSS platform framework and incorporated social sensors and CPS nodes into the CPSS platform to address collaborative and personalized production under the social manufacturing environment. Wang and Yang [25] clarified the data fusion in CPSS and proposed tensor-based methods and a framework for CPSS. However, this research focused on the data-centric perspective and seldom investigated the interaction of tri-spaces.

As for the applications of CPSS, Frazzon and Hartmann [15] reviewed the social aspects of CPS and introduced social–CPS (SCPS) application in production networks. Three context-dependent behavioral aspects were identified, including the individual, organizational and contextual backgrounds. Zhang and Yu [16] presented a CPSS with parallel learning for the distributed energy management (DEM) of a microgrid. Dao and Pongpaichet [28] proposed a real-time complex event discovery platform for CPSS. Gang and Fenghua [26] introduced ACP methodology (i.e. artificial societies, computational experiments and parallel execution), proposed by Wang, and discussed a CPSS-based intelligent transportation system (ITS). Zhang and Wang [7] discussed the state-of-the-art of ACP-based CPSSs in China with application in transportation, energy, manufacturing, etc. and five grids, including transportation grid, energy grid, information grid, the Internet of Things and the Internet of Minds. Zhong and Dong et al [29] proposed a CPSS for command and control, and described the self-synchronization and operational mechanism. Leng and Jiang [30] introduced the social Internet of Things (SIoT) strategy to reduce the complexity of contextual computing in the cyber–physical–social connected space. However, these were regarding specific applications in a certain field; few applications leveraged the data-driven methodologies.

### 2.3. Social Manufacturing

The manufacturing paradigm evolved from craft manufacturing to mass production, to mass customization and now to social manufacturing [2]. Jiang [31] introduced the concepts, architecture and key enabling technologies of the social manufacturing paradigm. Social manufacturing was first proposed by The Economist in 2012. Jiang and Leng [32] introduced the concepts and characteristics of social manufacturing. Social manufacturing has three characteristics, namely, social-oriented interconnection, service-oriented transformation, and IoT-oriented production structure [33]. Social manufacturing was viewed as cyber–physical–social connected and service-oriented. Ding and Jiang [34] proposed social sensors for social manufacturing systems (SMS) to address the production interactions among stakeholders in SMS, covering the concept, components, classification, operational logics, formalization and social sensor-cloud platform. However, social sensors mainly focused on data sensing and information exchange; the process of data analysis was missing. Jiang and Ding [35] introduced the definition and organizational logic of social manufacturing. In that work, three core aspects of social manufacturing were illustrated from the perspectives of configuration, Industry 4.0-based production control and business collaboration. However, the interactions among the cyber space, social space and physical space were not explored. Zheng and Xu [36] proposed a data-driven cyber–physical approach for personalized smart, connected product (SCP) co-development in a cloud-based environment. However, their research focused on the user–designer interaction based on data-driven cyber–physical systems. As the social manufacturing paradigm is still in its early stage of development, most existing literature concentrates on the concepts, architecture, enabling technologies, social networks and social collaboration based on cyber–physical systems. However, there were few works investigating the interaction between social space and CPS in the context of social manufacturing. There are few works on CPSS supporting social manufacturing.

### 2.4. Research Gap

The existing literature shows that D-CPSS is still in its infancy; few existing works focused on the interaction among tri-space. Although a data-centric framework for CPSS was proposed, the framework was conceptual and the interactions among the tri-space were missing. Most existing works focused on CPSS supporting smart manufacturing or wisdom manufacturing; however, few literatures discussed CPSS in the context of social manufacturing. Social manufacturing is named as such for its inclusion of the interaction and interdependence of social space, physical space and cyber space. In most research works, the social space in CPSS referred to humans as individuals, not in social networks. The social space component and collective intelligence were seldom investigated under the social manufacturing environment.

A large volume of data is generated from social space, cyber space and physical space. To this end, a data-centric view is necessary to link the tri-space. However, in terms of the data-driven approach, there is a lack of systematical investigation on the integration of CPSS and the data–information–knowledge–wisdom (DIKW) in the context of social manufacturing, although a data-centric framework for CPSS and data processing along the DIKW pyramid already existed. To address the interaction issues among the tri-space, this paper proposed a 7C framework for D-CPSS in the social manufacturing context from the perspective of the data–information–knowledge–wisdom pyramid.

## 3. A 7C Level Framework for D-CPSS

### 3.1. 7C Model for CPSS

**Figure 2:**
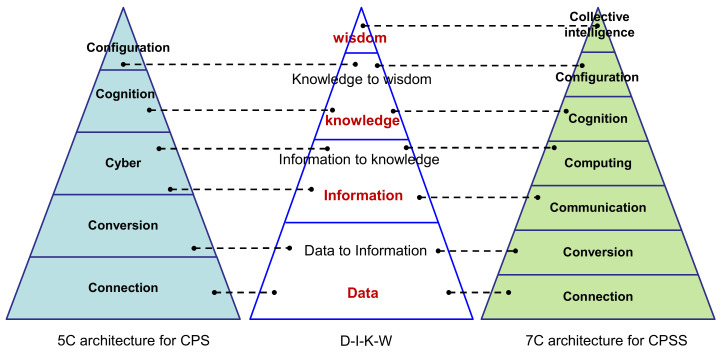
7C level model for CPSS.

Inspired by Lee, Bagheri and Kao 2015 [8], who proposed a 5C level structure for CPS, a 7C model for CPSS was put forward in this paper, including connection, conversion, computation, cognition, configuration and collective intelligence. Following the philosophy of the DIKW (data–information–knowledge–wisdom) pyramid, a schematic representation of the relationships among 5C model for CPS, DIKW and 7C model for CPSS is shown in Figure 2.

Presented above are three pyramids. In the middle lies the DIKW pyramid, which consists of seven levels, namely, the data level, data-to-information level, information level, information-to-knowledge level, knowledge level, knowledge-to-wisdom level and wisdom level. The data level is responsible for gathering data from different space. For example, sensors sense the context in the usage stage of the user and transmit product data and environmental data to information systems. The data-to-information level is responsible for convert that data into useful information. For example, data analysis tools are used to figure out the hidden information of user habits or the relationships of different data. The information level is responsible for information sharing between different spaces to facilitate communication so that user behaviors can be understood. The information-to-knowledge level deals with the generation of knowledge. For example, useful user information is often computed to form knowledge. The knowledge level is responsible for shaping of cognition. For example, user knowledge sharing on the web deepens users’ understanding of products and services. The knowledge-to-wisdom level is responsible for intelligence generation. For example, the interaction between smart products and users makes it possible for the product to make decisions via learning and training. The wisdom level means the ability to collect, process and share knowledge to generate solutions. For example, different cognitions enable the smart product to self-adapt to the change of context.

The connection relationship between 5C, DIKW and 7C is as follows: Both connection levels in the 5C and 7C frameworks mean data acquisition. Both conversion levels in the 5C and 7C frameworks correspond to the data-to-information level in the DIKW pyramid. The cyber level in the 5C framework and the communication level in the 7C framework correspond to the information level in the DIKW pyramid. The cyber level in the 5C framework and the computation level in 7C correspond to the information-to-knowledge level in the DIKW pyramid. Both cognition levels in the 5C and 7C frameworks correspond to the knowledge level in the DIKW pyramid. Both configuration levels in the 5C and 7C frameworks correspond to the knowledge-to-wisdom level in the DIKW pyramid. The collective intelligence level in the 7C framework corresponds to the wisdom level in the DIKW pyramid.

Figure 2 shows that the seven levels in the DIKW pyramid correspond to the seven levels in the 7C model one by one. From this perspective, the 7C model can be seen as a data-driven model. A detailed description of the 7C model is presented in the following subsections.

A comparison between the 5C model for CPS and the 7C model for CPSS is shown in Table 3. The results show that the cyber space in the 5C model is divided into communication and computation in the 7C model. More importantly, the 7C model emphasizes collective intelligence, which is the top level of the 7C model. Compared with the 5C model for CPS, the 7C model is designed for CPSS; the elements of the 7C model consider the social space. For example, the connection level collects data via social sensors and physical sensors. The conversion level converts social data into meaningful information. The communication level contains social networks. The computation level computes social information. The cognition level generates more social knowledge. The configuration level generates feedback from the cyber space to the social space and feedback from the physical space to the social space. The collective intelligence level emphasizes the synergy effect of intelligence.

#### 3.1.1. Connection

This level refers to connection of human or things in D-CPSS to support data acquisition from the perspective of the Internet of Everything (IoE), such as the Internet of People (IoP), Internet of Things (IoT), Internet of Content (IoCon), Internet of Computing (IoCom), Internet of Service (IoS), Internet of Thinking (IoTk) and Internet of Minds (IoM). Data are the raw materials for a data-driven CPSS. As opposed to CPS, the data source of CPSS comes from three sources: social space, physical space and cyber space. Each space exhibits interconnected features. In the social space, the connection of people forms social networks. In the physical space, the connection of things forms networks, such as sensor networks and machine networks. In the cyber space, the connection of computing forms computing networks. Data can be acquired via social sensors, physical sensors and information systems databases.

#### 3.1.2. Conversion

This level deals with converting data into information, aiming to discover correlation and a hidden pattern from the heterogeneous data to extract useful information. Data are gathered and stored in different databases depending on different purposes. Heterogeneous data from social sensors and physical sensors can be sorted into three types, namely, structured data, semi-structured data and unstructured data. A distributed database system (DDBS) is responsible for managing and storing the structured data and a Hadoop distributed file system (HDFS) or not only structured query language (NoSQL) are used to store unstructured data, while semi-structured data are unified into a standardized format by extensible markup language (XML) and stored in DDBS or relational database management system (RDBMS). Distributed data resources need to be collected to discover useful and correlative information and converted into actionable information.

The main tools and methodologies of this level concern data mining. Data mining techniques (DMTs) are used to search for hidden information in a large volume of data in the data mining processes. Data mining methods include clustering, association, classification and regression. For example, deep learning, Bayesian learning and clustering are the most common algorithms at this level.

#### 3.1.3. Communication

This level relates to information exchange and sharing for bridging the social space and the cyber space and physical space. From the viewpoint of interaction objects, communication can be divided into three types, including human-to-human, human-to-machine, and machine-to-machine. In the social space, the communication relationship is human-to-human via social networking or social media to support communication among customers, users, engineers, designers and suppliers. In the physical space, the communication relationship is machine-to-machine. Between the social space and physical space, the communication relationship is human-to-machine via human–machine interface. Algorithms such as communication protocols, human-in-the-loop learning and human-agent interaction play important roles at this level.

#### 3.1.4. Computation

The computation level is the core of this framework. Computation refers to a series of processes to convert information to knowledge and gain insights. Cloud computing and data analytics play significant roles in this level. Storm computing frameworks are used for processing real-time data and a Hadoop computing framework is used to process non-real-time data. Data analytics are used to describe what happened, diagnose why it happened and predict what will happen.

#### 3.1.5. Cognition

The cognition level is responsible for knowledge sharing. After generating knowledge in the computation level, the knowledge flows into the social space and physical space. As for social space, certain knowledge, such as expert knowledge or crowdsourcing, is used for generating insights. In the physical space, knowledge automation enables smart devices to have the capabilities of self-awareness, self-adaption, self-learning and self-decision making. In both the social and physical space, the cognition level plays the role of the Internet of Thinking (IoTk). Algorithms such as social cognition and interaction, structural learning and knowledge capture play important roles at this level.

#### 3.1.6. Configuration

Similar to the configuration level in 5C for CPS, this level is the feedback and control from the cyber space to the social space and physical space. The main objective of this level is to convert knowledge to insight for decisions to be taken, including descriptive insights, diagnostic insights, predictive insights and prescriptive insights.

#### 3.1.7. Collective Intelligence

This level refers to social collective intelligence in the social space and machine intelligence in the physical space. After data processing in computation level, the data from the tri-space will be fused for collective intelligence. Decisions are made in a co-creation mechanism. In the social space, decisions are made based on social collective intelligence via the Internet of Wisdom (IoW). In the physical space, decisions are made according to machine intelligence via the Internet of Intelligence (IoI). Machine learning algorithms play important roles at this level, including evolutionary learning, transfer learning, adaptation learning and multi-task learning.

### 3.2. D-CPSS

This subsection gives an introduction of D-CPSS and a general model for D-CPSS.

#### 3.2.1. The Components of D-CPSS

Similar to the ubiquitous CPSS, as shown in Figure 1, the components of the D-CPSS include the social space, physical space, and cyber space. The overlapped sections are the cyber–social system (CSS), cyber–physical system (CPS) and social–physical system (SPS). The components are shown in Table 4.

Cyber space. Cyber space is the Internet of Service (IoS). It provides data sensing, resource management and computing service. Big data processing is a key element in this space. Data processing in computation aims at conversion, including data-to-information, information-to-knowledge and knowledge-to-wisdom.

Physical space: Physical space means the real world to be monitored or controlled. It is made up of physical entity (such as equipment, devices, etc.) and interface device (such as sensors, actuators and controllers) to support interaction with cyber space and social space.

Social space: Social space refers to human society full of thinking, cognition, knowledge and collective intelligence. The social network or social media platform integrates stakeholders such as customers, users and designers to conduct social communication and social collaboration.

Cyber–social system (CSS): CSS connects the cyber space and social space via social sensors network. Social computing is used to analyze the social data.

Cyber–physical system (CPS): CPS connects the cyber space and physical space. A physical sensors network provides data for computing, monitoring and control of the physical devices.

Social–physical system (SPS): SPS connects the social space, or the Internet of People (IoP), and the physical space, or the Internet of Things (IoT), to facilitate the interaction between human and machine.

#### 3.2.2. A General Model for D-CPSS

**Figure 3:**
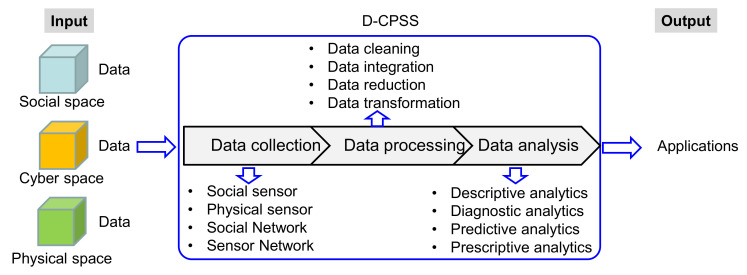
A general model of D-CPSS from the perspective of data chain.

A general model of D-CPSS from the perspective of the data chain is presented in Figure 3. As the input of D-CPSS, heterogeneous data sources initially form the social space, physical space and cyber space. The output of the D-CPSS is data applications. D-CPSS has three core functions, including data collection, data processing and data analytics. These processes will be elaborated in details as follows.

(1) Data collection from tri-space

The data are collected from three spaces (i.e., social space, cyber space and physical space) via four ways, namely social sensors, physical sensors, social network and sensor network.

Social sensor: Social sensors are responsible for measuring human social data, such as psychological data, emotional data, behaviors and comments.

Physical sensor: Physical sensors detect the data from physical devices (i.e., sensor, actuators and processors) and information systems. The whole lifecycle data are monitored and captured based on the configurations of data sensing devices (e.g., smart sensors, embedded devices, tags, RFID readers and external devices) through the whole lifecycle.

Social networks: Social networks are the Internet of People, or Social IoT, which connects people, data and collective intelligence.

Sensor networks: Sensors are interconnected via the Internet of Things (IoT), forming a sensor network, including a physical sensor network and a social sensor network. Distributed data resources are gathered together. They are multi-source heterogeneous big data, including equipment data, production data, products data, user data, supply chain data and so forth.

The collected data are stored both in the cloud datastore and edge database. The collaboration of cloud computing and edge computing makes it possible to solve such a large dataset with a particular focus on the application requirements. For low-latency application requirements, the data are stored and computed on the edge. For other application requirements, the data are managed in the cloud.

(2) Data processing

Among the captured data, there are large amounts of redundant, misleading, duplicate and noisy data which need to be removed before data analysis. Data processing refers to the process of converting data into information, which includes data cleansing, data integration, data reduction and data transformation.

- Data cleaning: This aims to identify and pad missing data, remove noisy data, wipe out isolated points and rectify data inconsistencies in order to enhance the quality of data.
- Data integration: This aims to store all the distributed data in a database or data warehouse to form a complete data set via removing redundant data.
- Data reduction: This aims to remove the attributes that cannot represent the key features of the system to reduce the data volume by ensuring that the reduced reflection of the data set are close to the original one, achieving the same or similar analysis results.
- Data transformation: This aims to transform the original data to the required format to satisfy the data mining requirement, for instance, by limiting the data value to a specific span. Common data transformation strategies include smoothing, aggression, normalization and discretization.

(3) Data analysis

Big data analytics can be divided into four types, namely, descriptive analysis, diagnostic analysis, predictive analysis and prescriptive analysis.

- Descriptive analysis is responsible for describing what happened.
- Diagnostic analysis is used to figure out why it happened when the performance degenerates or a failure occurs.
- Predictive analysis is used to predict what will happen in the future and the likelihood of a situation occurring by utilizing models with real time data or historical data.
- Prescriptive analysis is about suggesting what action to take and for decision-making to solve the problem.

### 3.3. A 7C Level Framework for D-CPSS towards Social Manufacturing

**Figure 4:**
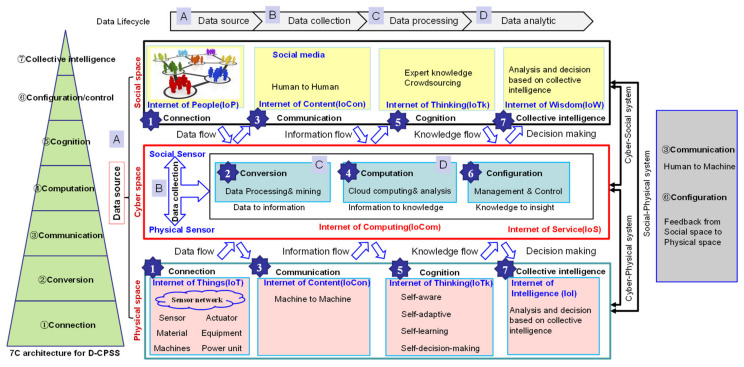
The 7C framework for D-CPSS towards social manufacturing.

By combining the general model for D-CPSS (see Figure 3) and 7C level model for CPSS (see Figure 2), Figure 4 depicts the 7C framework for D-CPSS under social manufacturing environment.

Each level in the 7C model for CPSS is combined with three spaces, i.e., the social space, cyber space and physical space. The connection level (level 1), communication level (level 3), cognition level (level 5) and collective intelligence level (level 7) are distributed in both the social space and physical space. The conversion level (level 2), computation level (level 4) and configuration level (level 6) are located in the cyber space.

Correspondingly, data flow, information flow and knowledge flow run in the social space and physical space. The conversion of data to information, information to knowledge, and knowledge to wisdom (insight) are accomplished in cyber space.

The data lifecycle consists of data source, data collection, data processing, data analytics and data application. The cyber–social system (CSS) supports the interaction between the social space and cyber space along the 7C level from connection to collective intelligence. Similarly, the cyber–physical system (CPS) supports the interaction between the cyber space and physical space along the 7C level from connection to collective intelligence. The social–physical system (SPS) supports the integration of the social space and physical space, focusing on communication and configuration.

## 4. Illustrative Example

### 4.1. Example Description

Company X is a smartphone manufacturer in China. It offers high-quality hardware and continuous software updates for users via open innovation or co-innovation. Customers and users are deeply involved in all stages of value co-creation, including design, development, testing and delivery. A large volume of data were generated from the social space and physical space during the usage of smart phones and complementary smart products. Facing the personalized needs of customers, Company X adopted social manufacturing by taking advantage of collective intelligence from the users, partners and designers. Artificial intelligence (AI) and the Internet of Things (IoT) were employed to enable an AI with IoT (AIoT) platform supporting the interaction of cyber space, physical space, and social space.

### 4.2. Application of 7C Framework for D-CPSS in Smart Product Development

**Figure 5:**
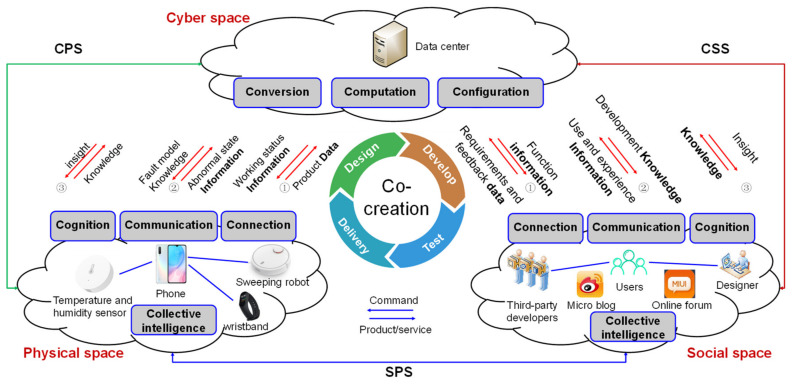
The application scenario of 7C framework for D-CPSS.

Figure 5 depicts the application scenario of D-CPSS in the value co-creation of smart products.

The loop in the center stands for the product development process, surrounded by the cyber space, physical space and social space. The product development process can be classified into four stages, namely, design, development, testing and delivery. The cyber space is responsible for conversion, computation and configuration. The physical space and social space deal with connection, communication, cognition and collective intelligence. The physical space is made up of smart and connected products, such as smartphones, wristbands, sweeping robots, temperature and humidity sensors, etc. The social space consists of users, designers, third-party developers, etc. The cyber–social system bridges the cyber space and social space, providing convenience for the exchange of data, information, knowledge and insight. Similarly, the cyber–physical system links the cyber space and physical space for the transfer of data, information, knowledge and insight. Social–physical space connects social space with physical space.

Connection: in the social space, the users, designers and third-party developers are interconnected via social media platform, such as online forum and micro blog. The connection is of high efficiency and low cost by taking advantage of the internet. Correspondingly, in the physical space, the smart devices are connected via the Internet of Things.

At the first stage, users communicate with the designers via a social media platform in social space. Key customers engage in the testing of software and offer feedback on the functioning of the version. User requirements data are transferred to the big data platform in the cyber space. Statistically, the users’ requirements for the product function show a long tail distribution, which means there is a high proportion of “head” user requirements. Although the proportion of long tail is low, there exists valuable information on user requirements, such as customization and personalization.

Conversion: Big data analytics are used to convert the requirement data in the social space to information on the product function and filter useful information from the massive user feedback. Then, the information on the functioning is transferred from the cyber space to the social space for the next stage of development. On the other hand, product data in the physical space are converted into product working status information. The information on product performance is transferred from the cyber space to physical space for assessing working status.

At the second stage, Company X focuses on the development of core functions and outsources the development of non-core functions to third-party developers or customers. This is based on the communication in the social space among the designers in Company X, users and third-party developers.

Communication: The designers and the users communicate closely and repeatedly to confirm the product function details in social space. If the function development is out of the capability scope of Company X, the designers will communicate closely with the third-party developers and outsource development tasks to third parties. In the physical space, the smart devices in use can send messages and information to the smartphone via the Internet of Things by machine-to-machine communication.

At the third stage, after the function is developed, Company X selects a large number of users for prototype testing. Users provide comments and suggestions for improvement after using the product in social space. Both the good and bad feedback on user experience information are transferred to the cyber space for computation.

Computation: All the problems that appeared during the testing period are investigated via data mining or machine learning algorithms in cyber space to figure out the root cause. Based on users’ good feedback, the development experience and approaches are summarized to form development knowledge for designers to share. The abnormal information (e.g., defect and faults) is sent to the cyber space for analyzing. The failure mode is generated by modeling and added to the knowledge library or model library.

Cognition: The knowledge on the development is shared in the social space, while the knowledge on the product is shared in the physical space. As for the social space, each participant updates their cognition, including the designers, third-party developers and users. Designers gain new design thinking from third-party developers. Third-party developers learn the development model and skills from designers, such as software development thinking for hardware development. As for the physical space, taking the sweeping robot as an example, it automatically plans routes based on the location of the trash after the self-learning and self-adaption.

Configuration: The cyber space provides feedback and control for the social space and physical space. As for the social space, this converts social cognition into insight on the market, supporting decision-making about when to launch the product. As for the physical space, this converts cognition into machine intelligence for self-decision-making.

Collective intelligence: In the social space, crowds make the decision on which function will be equipped on the final product to be launched to the market. In the physical space, taking smart home devices as an example, the temperature and humidity sensor sensing the indoor environment, the air conditioning automatically adjusts the temperature according to a sensor.

At the last stage, the product is released on to the market. The users realize their ideas by engaging in the whole process of development and witness their personal requirements coming into fruition. With social participation and the interaction with the physical and cyber space, the data in the tri-space is utilized to generate value.

### 4.3. Results

The data-driven thinking integrated the social space, physical space and cyber space. The information, knowledge and wisdom (i.e., collective intelligence) were exchanged in an accelerating flow. The CPS, CSS and SPS facilitated the data processing and data analysis for decision-making. After the implementation of the 7C level framework for D-CPSS, the direct effects were a shorter cycle time of smart product development, more precise and appealing functions to meet personalized needs, reduced cost of the development and higher interaction frequency among social space, cyber space and physical space. The indirect effects were embodied in three aspects, including the users’ viscosity to the products, more partners providing complementary products or components for the platform and more users’ involvement in the co-creation process.

## 5. Discussion

This section discusses theoretical implications, practical implications, limitations and technical challenges.

### 5.1. The Theoretical Implications

The proposed framework in this paper has some advantages in comparison with other previous frameworks, as shown in Table 5.

A seven-layered framework for D-CPSS is presented from a data-driven perspective. This paper provides theoretical basis for future research on data-centric CPSS. The theoretical implications of this paper are as follows:

(1) The proposed 7C model for CPSS contributes to the field of CPSS by extending the scope of the 5C level framework for CPS. The specific meaning of each level is different from that of 5C, because social space is considered in each level. The proposed framework deepens the understanding of data-driven CPSS by presenting the process from data to collective intelligence. It provides new directions for the future research on D-CPSS. For example, the AI model selection in each level will affect the efficiency and effectiveness of each level and the final results of D-CPSS.
(2) The data–information–knowledge–wisdom logic can be used in other fields concerning about data-centric or data-driven complex systems. The DIKW pyramid can be adopted in the development and implementation of smart systems that have machine intelligence.
(3) The combination of 7C level model, general data-driven model and CPSS contributed to the generation of the proposed 7C level framework for D-CPSS, which is a system of systems. Collective intelligence, as the top level of 7C, emphasizes the power of the crowd. More social value will be generated based on the interactions among the tri-space. Social factors are increasingly important.

### 5.2. The Practical Implications

The proposed framework can be used in practice under the environment of social manufacturing, which is service-oriented and user-centric. The practical implications are as follows:

(1) The proposed framework can offer references for the transformation from CPS-based smart manufacturing to CPSS-based social manufacturing. It can be applied to guide the development of other smart products, such as smart household electrical appliances and smart vehicles.
(2) Humans on a societal level will benefit from the 7C solution. For example, the Internet of Thinking, Internet of Wisdom and Internet of Communication facilitate the sharing, fusion and generation of knowledge. Compared with previous approaches, the top level of the 7C model (i.e., collective intelligence) will generate new social values in the use stage of users, such as new collaborative information generating from the interaction between human and machine for decision-making. The proposed framework can provide references for human–machine interaction and collective intelligence of machine.

### 5.3. Limitations and Technical Challenges

The D-CPSS can be seen as a system of systems, as it contains CPS, CSS and SPS. The limitation of this paper is that the interactions among these systems themselves were not investigated. The implementation of the framework relies on big data analysis and AI models. How to choose suitable analysis tools and algorithms for each level is a technical challenge that requires further exploration throughout the process of transformation from data to collective intelligence. Especially, the collective machine intelligence in physical space depends on a large number of algorithms.

## 6. Conclusions and Future Perspective

In this paper, we proposed a 7C framework for D-CPSS from a systematic perspective, including connection, conversion, communication, computation, cognition, configuration and collective intelligence. Originating from CPS, the evolution and components of CPSS were introduced. We combined the 7C model with CPSS from a data-centric view and presented the integrated framework of 7C level D-CPSS for social manufacturing. A general model of D-CPSS from the perspective of the data chain was proposed. An illustrative example of Company X for product co-creation was given to examine the framework.

The main contributions of this paper to the field of CPSS can be summarized as follows:

(1) A 7C model for data-driven CPSS along the data–information–knowledge–wisdom (DIKW) pyramid was proposed, inspired by the perspective of data lifecycle: data source, data collection, data processing, data analysis and data applications. On the one hand, with connection, communication, cognition and collective intelligence distributed in both the social space and physical space, and conversion, computation and configuration located in the cyber space, the 7C model integrated the tri-space of CPSS. On the other hand, corresponding to the seven levels in the DIKW pyramid, (i.e., data level, data-to-information level, information level, information-to-knowledge level, knowledge level, knowledge-to-wisdom level and wisdom level), the 7C model was data-driven. Therefore, the combination of these two aspects supported the 7C model for data-driven CPSS.
(2) A general model for D-CPSS was proposed, including input, output and processing. The processing ranged from data collection to data processing and data analytics. From the perspective of system engineering, D-CPSS was seen as the processing block. To support social manufacturing, a large volume of data-based applications was generated via D-CPSS. Data from cyber space, physical space and social space were the data source of D-CPSS.
(3) We proposed a 7C framework for D-CPSS under a social manufacturing context. Interactions among the social space, cyber space, and physical space were detailed. Compared with CPS and traditional CPSS, D-CPSS under social manufacturing emphasized the importance of social factors and being data-driven. Level 1, level 3, level 5 and level 7 in the 7C model are distributed in both the social space and physical space; level 2, level 4 and level 6 in the 7C model are located in the cyber space. The interactions among the tri-space of D-CPSS were along the DIKW pyramid.

As D-CPSS is promising and has great potential in social manufacturing, there are challenges that need to be addressed in the future research as follows.

(1) Privacy and security of D-CPSS is a big challenge, including, for example, data protection for user authentication [37]. With the development of the Internet of Things (IoT), an increasing amount of devices and humans are involved in the CPSS; the privacy protection and cyber security of users is a future research direction. Blockchain-based mobile-edge computing is promising to address this challenge [38].
(2) D-CPSS is still in its infancy stage; especially, machine intelligence requires much more research work. With the development of Artificial Intelligence technologies, the devices will be much more intelligent and autonomous. The interaction and interoperability between the physical space and social space is a direction for future research.
(3) The CPSS can be viewed as an ecosystem, consisting of social space, physical space and cyber space. The actors in the CPSS are heterogeneous. The manufacturing resource sharing mechanisms, such as the knowledge sharing mechanism in D-CPSS, call for deeper investigation to guide the operation of CPSS.

## Figures and Tables

**Table 1:** Definition and research on the cyber–physical–social system (CPSS).

Concept	Literature	Definition
Socio–cyber–physical systems (SCPS)	[14,15]	The extension of CPS by adding social space.
Cyber–physical–social systems (CPSS)	[8,13,16,17]	A complex system involving the cyber, physical and social space by integrating CPS with human and social characteristics.
Human–cyber–physical systems (HCPS)	[11,12]	The evolution of the manufacturing system, which is described as human–physical–system (HPS), by adding cyber systems.
Human-in-the-loop cyber–physical systems (HiL-CPS)	[6]	CPSs include humans as an integral part in the control loop.
Cyber–physical–social–thinking systems (CPST)	[18]	A hyperspace created by merging thinking space with the cyber space, physical space and social space.

**Table 2:** Literature on CPSS.

Dimensions	Examples of Literatures	Contribution	Methods
Definition and concept	[4]	Definition of CPSS as a system of systems, including CPS, social systems, virtual space and physical space.	Review
	[20]	Systematic review on CPSS, including concepts, characteristics, process and applications.	Systematic mapping study
	[18]	Cyber–physical–social–thinking (CPST) hyperspace architecture.	Conceptual framework
	[21]	Socio–cyber–physical system (SCPS)-based manufacturing.	Conceptual framework
Architecture	[8]	Data-centric framework for CPSS.	Conceptual framework
	[14]	An architecture of social–CPS (SCPS)-based manufacturing.	Conceptual framework
	[22,23]	A three-layer CPSS platform framework under the social manufacturing environment.	Conceptual framework
Design	[17]	A survey on design methodology for CPSS.	Survey
	[24]	Petri net-based extended model for CPSS.	Petri net
	[25]	Data fusion in CPSS.	Tensor based methods
Applications	[15]	Social–CPS (SCPS) application in production networks.	Review
	[16]	CPSS with parallel learning for distributed energy management (DEM) of a microgrid.	Parallel learning methodology
	[26]	CPSS-based intelligent transportation system (ITS).	Artificial societies, computational experiments and parallel execution (ACP) methodology
	[7]	ACP-based CPSS applications in transportation, energy, manufacturing, etc.	Survey

**Table 3:** Comparison of components between the proposed 7C for CPSS and the existing 5C for CPS.

Components of 5C for CPS	Description	Components of 7C for CPSS	Description	Role
Level 1: Connection	Data acquisition from machines or sensors.	Level 1: Connection	Data acquisition form social sensors and physical sensors.	Data flow
Level 2: Conversion	converting data to information,	Level 2: Conversion	Converting data to information	Data to information
Level 3: Cyber	Analytics for insight	Level 3: Communication	Network	Information flow
Level 4: Cognition	knowledge generation	Level 4: Computation	Cloud Computing	Information to knowledge
Level 5: Configuration	feedback and control, decision	Level 5: Cognition	knowledge discovery	Knowledge flow
		Level 6: Configuration	Feedback and Control	Knowledge to insight
		Level 7: Collective intelligence	co-creation, the output of crowding	Insight for decision making

**Table 4:** The components of D-CPSS.

Space/System	Elements	Data	Internet of X
Cyber space	Data center, information system, software, cloud computing, big data	IoT big data	Internet of Computing, Internet of Services
Physical space	Physical entity (equipment, devices, tools), physical sensors, actuators, controllers	State of devices (temperature, humidity, electricity, time, location), environment	Internet of Things, Internet of Content, Internet of Thinking, Internet of Machine Intelligence
Social space	Social entity (customers, users, designers, developers), social network (social media), social sensors (smartphones, smart mobile device), crowdsourcing	Social data (demands, satisfaction, advice, feelings, comments, behaviors)	Internet of People, Internet of Content, Internet of Thinking, Internet of knowledge, Internet of Minds, Internet of Human Intelligence
Cyber–social system (CSS)	Social sensor network	Social data	Human-to-human interaction
Cyber–physical system (CPS)	Physical sensor network	Physical data	Internet of Things, machine-to-machine interaction
Social–physical system (SPS)	Product service system, human–computer interaction	Social data, physical data	Human-to-machine interaction

**Table 5:** The comparison of the proposed framework with other framework.

Literature	[9]	[8]	This Paper
Framework	5C framework for CPS	Six-layered framework for D-CPSS	7C framework for D-CPSS
Data centric	N/A	√	√
CPS	√	√	√
CPSS	N/A	√	√
D-CPSS	N/A	√	√
Interaction among social, physical, cyber space	N/A	N/A	√
D-I-K-W	Data flow, information flow	N/A	√
Smart manufacturing	√	√	√
Social manufacturing	N/A	√	√
Collective intelligence	N/A	N/A	√
